# Pharmacist knowledge of gout management: impact of an educational intervention

**DOI:** 10.1186/s41927-022-00259-x

**Published:** 2022-05-23

**Authors:** Emma R. Dorris, Mariosa Kieran, Nicola Dalbeth, Geraldine McCarthy

**Affiliations:** 1 UCD School of Medicine, University College Dublin, Dublin, Ireland; 2 Pharmacy Department, Mater Misericordiae University Hospital, Dublin, Ireland; 3 Department of Medicine, University of Auckland, Auckland, New Zealand; 4 Department of Rheumatology, Mater Misericordiae University Hospital, Dublin, Ireland

**Keywords:** Gout, Crystal arthritis, Multi-disciplinary care, Education

## Abstract

**Background:**

Pharmacists play a key role in community gout education. We investigated pharmacist knowledge of gout management and developed an educational intervention which was assessed in a cohort of Irish pharmacists.

**Methods:**

A ten-question questionnaire about gout management was developed to assess pharmacists’ knowledge. A 14 min 26 s video educational intervention was co-designed by a rheumatologist, a pharmacist, and designer of pharmacy education resources. The effectiveness of this pharmacy-specific intervention was assessed using the same questionnaire in 53 pharmacists (25 in the intervention group; 28 in the control group). Contingency tables were used to analyse differences between groups.

**Results:**

There were 173 pharmacist respondents to the initial survey; 35.3% answered that first-line therapy for gout involves a combination of a xanthine oxidase inhibitor (e.g., allopurinol) combined with a prophylactic agent (e.g., colchicine), and 28.9% of respondents answered that colchicine prophylaxis should be used when initiating urate-lowering therapy. Following the educational intervention, pharmacist’s knowledge about gout management increased across many domains, including serum urate targets when using urate-lowering therapy (*p* = 0.006), use of colchicine prophylaxis (*p* = 0.011), and duration of colchicine use (*p* < 0.001).

**Conclusion:**

Gout management recommendations can be impeded if translation into pharmacy practice is neglected. Pharmacists are a valuable information resource for patients. Co-designing a brief education intervention with pharmacists is an effective, low-cost way to increase pharmacist knowledge on the management of gout.

**Supplementary Information:**

The online version contains supplementary material available at 10.1186/s41927-022-00259-x.

## Key points

- The role of the pharmacist is critically important for the interprofessional management of gout, but pharmacists may not be aware of current recommendations for effective gout management.
- Low-cost education intervention is successful in increasing pharmacist knowledge about gout management.
- Recommendations for the management of gout, such as the 2020 American College of Rheumatology Guideline for the Management of Gout, should be targeted for knowledge translation into the pharmacist community.

## Background

Gout is the most prevalent form of inflammatory arthritis and associated with multiple serious comorbidities [1]. Effective management of gout is important to improve the negative impact the pain and inflammation has on patient quality of life. Evidence-based management guidelines have been published for the management of gout[2, 3]. Yet, adherence to therapy has been persistently low amongst gout patients. A recent meta-analysis estimated an overall adherence rate to urate-lowering therapy (ULT) of 47% [4].

Unlike many other forms of arthritis, the pathobiology of gout is well understood [5]. Hyperuricaemia, due to an underexcretion and/or and overproduction of urate, leads to monosodium urate (MSU) crystal formation and deposition in joints and tissues in susceptible individuals. Acute gout flares occur as an inflammatory response to MSU crystals. The goal of urate lowering therapy is to reduce serum urate (sUA) to a therapeutic target level, thereby permitting crystal dissolution as well as preventing further crystal formation and deposition. The rate of crystal reduction is dependent upon both the total crystal load and reduction in sUA. Gout flares can be precipitated by the introduction of ULT and can continue to occur for months after the therapeutic target sUA has been reached [6]. Thus, an anti-inflammatory prophylaxis, such as colchicine, is recommended for the at least the first three to six months following initiation of ULT [3]. Otherwise, the likelihood of discontinuation of ULT is substantially increased.

Pharmacists are often the most easily accessible members of a patient’s health care team. Pharmacists are a key source of information on disease management for patients, and patients have confidence in them [7]. They are, therefore, critical resources for effective management of disease, particularly when chronic. This is exemplified by the Randomized Evaluation of an Ambulatory Care Pharmacist-Led Intervention to Optimize Urate Lowering Pathways (RAmP-UP) study, a randomized controlled trial which demonstrated pharmacist-led intervention resulted in significant improvements in gout care [8]. However, that study also demonstrated that these effects were attenuated over time and highlighted the importance of additional efforts to enhance patient engagement in gout management.

The catalyst for this project was patients describing to their rheumatologist (GMcC) that they were being given conflicting advice from their pharmacists about gout management, particularly in relation to colchicine use. This led to a project investigating the knowledge of pharmacists about gout management, and subsequently, the development of an education intervention designed specifically for pharmacists.

## Methods

### Ethics

This study was deemed exempt from ethical review under the Mater Misericordiae University Hospital and Mater Private Hospital Institutional Review Board.

### Survey development

A ten-question questionnaire about gout management was developed to assess pharmacist knowledge in line with survey design principles as per Kelley[9]. Survey readability was reviewed by five healthcare professionals, including two pharmacists. Survey Monkey was used as the survey platform. Only nominated members for the project team had access to response data. An open format survey was used, with participants self-declaring if they were pharmacists. All questions were optional excluding question one (Where do you practice?) which was mandatory. The survey was only available in the English language.

### Sampling and survey administration

Non-random sampling was used. The survey was advertised via the Pharmaceutical Society of Ireland (the national Pharmacy Regulator) newsletter (October 2018), via pharmacist word of mouth (December 2018–Jan 2019) and posted onto a Pharmacist-Only community forum (Pharmabuddy) March 2019. There was 100% completion rate per question, with no skipped answers.

### Intervention development

The education intervention was co-developed by two consultant rheumatologists and gout specialists, a pharmacist and general practitioner. The information contained in the 14-min video was based on the 2016 EULAR gout management guidelines [2]. The survey aligned well with the EULAR guidelines as they are appropriate for Ireland as well as the rest of Europe. The format of the video was designed to maximise viewership. Based on information about pharmacists’ use of the eLearning platform Pharmabuddy, the video was divided into 11 discrete sections on gout topics, bookended by an introduction and close from the site founder (video available in Additional file 1). These discrete sections were designed to last less than 130 s each. This format allowed each key point to be a focus, with the goal of facilitating compound learning.

### Intervention assessment

The survey was posted onto the eLearning platform, with an additional question as to whether the pharmacist had viewed the education video. This version of the survey was only available via the eLearning platform between January 2020 and February 2020. The intervention video had been launched nine months prior. Pharmacists self-selected to participate in the survey.

### Data analysis

Log files were checked for duplicate entries. Data was analysed in IBM SPSS v24. Data were analysed with contingency tables for association between groups. Due to sparse responses in some categories, statistical tests comprised Fisher's exact test (for 2 × 2 tables), or Pearson's chi-square test with p-values derived from 10,000 Monte Carlo permutations (for larger contingency tables). A p-value of < 0.05 was considered statistically significant.

## Results

### Study participants

At yearend 2018, there were 6220 registered pharmacists in Ireland [10]. There were 173 respondents to the survey, equivalent to 2.78% total pharmacists in Ireland. Of the 173 pharmacists who responded to the survey, 155 (89.6%) were community-based pharmacists. Pharmacists practicing in all four provinces of Ireland responded, however, pharmacists practicing in the province of Ulster were underrepresented at only 1.7% (*n* = 3).

### Initial survey results

The frequency of responses can be found in Table 1. Pharmacist knowledge about gout management was not aligned with the 2016 EULAR gout management guidelines. Of note, consistent with the anecdotal reports from patients, 117 (67.6%) of pharmacists were not aware that patients are advised to take colchicine continuously for six months or longer after initiation of ULT. In addition, 109 (63.0%) did not know that colchicine twice daily was used as a prophylaxis for gout flares. When asked what source of information most pharmacists referenced for colchicine information, the overwhelming majority used the British National Formulary (BNF; used by 85.5% of respondents) or the summary of product characteristics (SPC; 57.8%) rather than rheumatology society guidelines such as the EULAR or ACR gout management guidelines (8.7%).

**Table 1:** Response frequencies from the pharmacists’ knowledge of gout management survey

Knowledge of gout management survey	Answer options	Frequency
Where is your primary area of practice?	Community	155 (90%)
	Hospital	14 (8%)
	Academia	1 (0.6%)
	Industry	1 (0.6%)
	Other	2 (1%)
Urate-lowering therapy (ULT) is targeted to serum urate level	True	126 (73%)
	False	17 (10%)
	Don’t know	28 (16%)
First-line therapy for gout involves a combination of a xanthine oxidase inhibitor (e.g., allopurinol) combined with a prophylactic agent (e.g., colchicine)	True	61 (35%)
	False	109 (63%)
	Don’t know	3 (2%)
Colchicine at a dose of 0.5 mg twice daily should be given in combination with urate lowering therapy (ULT) for at least 6 months after initiation of ULT, as a prophylaxis for gout flares	True	50 (29%)
	False	109 (63%)
	Don’t know	14 (8%)
It is common for gout to flare when..	Starting ULT	66 (38%)
	Stopping ULT	28 (16%)
	Increasing ULT	3 (2%)
	All of the above	64 (37%)
	None of the above	12 (7%)
Patients should stop ULT during a gout attack	True	42 (24%)
	False	116 (67%)
	Don’t know	15 (9%)
Patients are advised to take colchicine continuously for six months or longer after initiation of ULT	True	39 (23%)
	False	117 (68%)
	Don’t know	17 (10%)
Patients should dose-reduce rather than stop colchicine if they experience side-effects (e.g., diarrhoea)	True	77 (45%)
	False	72 (42%)
	Don’t know	24 (14%)
Which information sources for colchicine do you find helpful?	BNF	148 (86%)
	SPC	100 (58%)
	Guidelines (e.g., EULAR or ACR)	15 (9%)
Cost of gout medication is a factor in patient non-adherence	Agree	40 (23%)
	Disagree	87 (50%)
	Neither agree nor disagree	46 (27%)
What county do you work in?	Munster	68 (39%)
	Dublin	38 (22%)
	Leinster (Excl Dublin)	33 (19%)
	Connaught	22 (13%)
	Ulster	3 (2%)
	Not specified	9 (5%)

### Effectiveness of the educational intervention

In response to the results of the survey, an education intervention was co-developed between a consultant rheumatologist, a community pharmacist, and the founder of a free pharmacist-only resource designed by pharmacists for pharmacists, with the aim of providing education and resources for continued professional development within the Irish pharmacist community. A video tutorial, lasting 14 min, 26 s, was developed. The topics covered are listed in Table 2. The video was subdivided into segments to facilitate quick and easy access and to act as a reference guide for pharmacists, past its use as an education tutorial.

**Table 2:** Education intervention: video segments

Topic	Duration
What is the acute treatment of gout?	1 min 14 s
In the treatment of gout, when would you choose one treatment over another?	1 min 09 s
When should urate lowering therapy be started in patients?	1 min 39 s
When would you choose allopurinol over febuxostat?	0 min 47 s
When would you choose febuxostat over allopurinol?	1 min 22 s
When starting urate lowering therapy, are there any points to be taken into consideration?	1 min 14 s
What is the evidence base for continuing regular colchicine in patients for 6 months after the initiation of urate lowering therapy?	1 min 52 s
What are the main interactions to be mindful of when starting urate lowering therapies?	0 min 37 s
Discuss the adverse effects of medications used in the acute management of gout	1 min 08 s
Discuss the adverse effects of urate lowering therapies	1 min 16 s
When should urate lowering therapies be discontinued?	2 min 08 s
Total duration	14 min 26 s

To assess the effectiveness of the education intervention, we used the same survey as before, with the addition of a question as to whether the respondent had watched the tutorial video. There were 25 respondents who had watched the video (intervention group) and 28 respondents who had not (control group). Those in the intervention group had significantly greater knowledge of gout management than the control group (Table 3). In particular, the intervention group had greater knowledge about colchicine and its use as prophylaxis following initiation of ULT. Furthermore, a greater proportion of the intervention group reported that they would use management guidelines, such as the EULAR or ACR guidelines for the management of gout, as a source of information (48.0% in the intervention group compared to 17.9% in the control, *p* = 0.037).

**Table 3:** Intervention assessment on pharmacists’ knowledge of gout

Intervention assessment survey			Control n (%)	Intervention n (%)	*p-value*
Where is your primary area of practice?		Community	25 (89%)	23 (92%)	0.74*
		Hospital	3 (11%)	2 (8%)	
Urate-lowering therapy (ULT) is targeted to serum urate level		True	19 (68%)	24 (96%)	***0.006***
		False	5 (18%)	1 (4%)	
		Don’t Know	4 (14%)	0 (0%)	
First-line therapy for gout involves a combination of a xanthine oxidase inhibitor (e.g., allopurinol) combined with a prophylactic agent (e.g., colchicine)		True	9 (32%)	16 (64%)	***0.011***
		False	17 (61%)	9 (36%)	
		Don’t Know	2 (7%)	0 (0%)	
Colchicine at a dose of 0.5 mg twice daily should be given in combination with urate lowering therapy (ULT) for at least 6 months after initiation of ULT, as a prophylaxis for gout flares		True	9 (32%)	21 (84%)	***0.001***
		False	17 (61%)	3 (12%)	
		Don’t Know	2 (7%)	1 (4%)	
It is common for gout to flare when..		Starting ULT	1 (4%)	7 (28%)	***0.053***
		Stopping ULT	4 (14%)	1(4%)	
		Increasing ULT	0 (0%)	0 (0%)	
		All of the Above	12 (43%)	17 (68%)	
		None of the above	1 (4%)	0 (0%)	
Patients should stop ULT during a gout attack		True	7 (25%)	1 (4%)	***0.10***
		False	20 (71%)	24 (96%)	
		Don’t Know	1 (4%)	0 (0%)	
Patients are advised to take colchicine continuously for six months or longer after initiation of ULT		True	10 (36%)	21 (84%)	***0.001***
		False	17 (61%)	4 (16%)	
		Don’t Know	1 (4%)	0 (0%)	
Patients should dose-reduce rather than stop colchicine if they experience side-effects (e.g., diarrhoea)		True	16 (57%)	18 (72%)	***0.24***
		False	11 (39%)	6 (24%)	
		Don’t Know	1 (4%)	1 (4%)	
Cost of medication is a factor in patient non-adherence		Agree	9 (32%)	6 (24%)	***0.36***
		Neutral	6 (21%)	6 (24%)	
		Disagree	13 (46%)	13 (52%)	
Which information sources for colchicine do you find helpful?	BNF	Yes	20 (71%)	18 (72%)	1.0*
		No	8 (29%)	7 (28%)	
	SPC	Yes	15 (54%)	14 (56%)	0.59*
		No	13 (46%)	11 (44%)	
	Guidelines such as EULAR or ACR	Yes	5 (18%)	12 (48%)	0.037*
		No	23 (82%)	13 (52%)	

*p-value calculated by Fishers’ exact two-tailed test. All other p-values calculated from 10,000 Monte Carlo permutations

## Discussion

Pharmacists, particularly community pharmacists, are a key member of the primary healthcare team and have a central role in providing education to patients about health conditions and medicines. It is estimated that more than 89% of the US population is within 5 miles of a pharmacy, making pharmacists one of the most easily accessed healthcare team members [11]. Thus, open communication and education between the pharmacy and prescriber communities is essential to provide the most up-do-date and appropriate patient care. However, pharmacists frequently report a lack of information and clinical connection to other healthcare professionals as barriers to providing optimal care to patients[11]. In this study we have demonstrated that pharmacists do not typically use disease management guidelines as standard sources of information, and as such, may not be up to date on the recommendations for gout management. This is somewhat understandable given the wide number of conditions encountered on a daily basis in community pharmacy and the wide number of society guidelines available. For a community pharmacist, it may be much more time efficient to refer to a generalised text such as the BNF or drug-specific information. As such, a dedicated effort must be made by prescribers and professional societies to communicate treatment standards to pharmacists.

The educational gaps for patients living with gout have been well explored in the literature. In addition to inaccurate common beliefs, patients typically report difficulty understanding the use of prophylaxis during the early phase of urate-lowering therapy and are often unaware of optimal treatment goals [12]. These knowledge gaps are not unique to patients and caregivers, education gaps exist for healthcare professionals as well. Here, we demonstrate that to be the case with pharmacists.

The BNF and SPC conveys clear information on colchicine use in the acute phase of gout flares. They also suggest that colchicine can be used for prophylaxis, but pharmacists report that this role of colchicine has been poorly communicated to pharmacy community and that clarity on duration of colchicine prophylaxis is lacking[13]. Furthermore, the phenomenon of legacy prescribing, whereby a short or intermediate-term medications are not appropriately discontinued, is prevalent [14]. Patients, and indeed pharmacists, therefore, may experience confusion as to whether the prescribed colchicine is indeed intended as a prophylaxis or is a legacy prescription that was previously prescribed in case of flare. This confusion can be confounded by lack of clear, definitive statements on updated best practices from national medicine information centre or similar national bodies.

In most countries, pharmacists must undertake continuing professional development (CPD) and typically are open to education that influences and benefits their professional practice [15]. In Ireland, the Pharmacy Act 2007 requires that all pharmacists in Ireland must undertake continuing professional development (CPD). This study acts as a proof of concept and the intervention presented here could be integrated into a CPD for gout management. This would have the benefit of increasing the reach of this information to pharmacists. An extended CPD could also incorporate other important areas for gout management that were beyond the scope of this study, such as lifestyle recommendations, medication selection and treatment of acute gout. We would recommend a co-design approach for this CPD between rheumatologists and pharmacists to ensure the most up to date knowledge is being disseminated to pharmacists in a manner most suited and accessible to practising pharmacists. A co-design approach could also improve the interest and uptake of a gout CPD module by pharmacists [16]. The onus is on the rheumatology community to communicate to pharmacists and other health care providers about up-to-date recommendations for gout management.

We have demonstrated that co-designing an education intervention with pharmacists can be an effective, low-cost way to increase pharmacist knowledge on the management of gout. In this study we assessed a single intervention. Of note, in our study a relatively high proportion of pharmacists that, despite having received the educational intervention, have insufficient knowledge about certain areas of gout management. The intervention was assessed approximately nine months after the training was released onto the pharmacist eLearning platform. The lag time between training and assessment was used to model the real world. However, spaced learning may be beneficial to reinforce education on gout management in the long-term. Thus, periodic review sessions with gout pharmacists on key gout management points could compound learning, although this has yet to be assessed. Distributed or spaced learning may be better for maintaining long-term knowledge, but other obstacles may prevent this approach from being applicable in a real-world scenario [17]. Given the breadth of the community pharmacist’s remit, there is seldom the incentive or feasibility to methodically revisit previously learned materials. This will need to be considered in follow up studies address this approach.

Strengths to this study include the co-design of the intervention with pharmacists and use of pharmacist-run learning platforms to maximise the accessibility of the intervention to the target audience. The limitations are the small sample size relative to the number of pharmacists registered in Ireland. This study focused on a specific issue encountered in practice. There are other relevant management issues related to gout management beyond the scope of this study. Thus, the survey results may not reflect all pharmacists’ knowledge.

## Conclusions

Gout is a well characterised disease with effective pharmaceutical intervention available. Yet, adherence to gout treatment is poor and the underlying reasons are complex. Education to improve gout management is typically focused on patients and doctors. Here we identify a gap in gout management within pharmacists, key providers of medical education to patients. We demonstrate that a short education intervention can increase pharmacist knowledge. These data also highlight the importance of open communication between rheumatologists and pharmacists if gout management recommendations are to be implemented in practice.

## Supplementary Information

**Additional file 1:** Tutorial video for pharmacists on the pharmacological management of gout.

## Data Availability

The datasets generated and analysed during the current study are available in the Figshare repository, https://doi.org/10.6084/m9.figshare.19307366.v2

## Abbreviations

ULT: Urate lowering therapy
MSU: Monosodium urate
sUA: Serum urate
RAmP-UP: Randomized Evaluation of an Ambulatory Care Pharmacist-Led Intervention to Optimize Urate Lowering Pathways
BNF: British National Formulary
SPC: Summary of product characteristics
CPD: Continuing professional development
